# The Use of Added Salt and Sugar in the Diet of Polish and Austrian Toddlers. Associated Factors and Dietary Patterns, Feeding and Maternal Practices

**DOI:** 10.3390/ijerph17145025

**Published:** 2020-07-13

**Authors:** Daria Masztalerz-Kozubek, Monika A. Zielinska, Petra Rust, Dorota Majchrzak, Jadwiga Hamulka

**Affiliations:** 1 Department of Human Nutrition, Institute of Human Nutrition Sciences, Warsaw University of Life Sciences (SGGW-WULS), 02-787 Warsaw, Poland; daria_masztalerz_kozubek@sggw.edu.pl (D.M.-K.); monika_zielinska@sggw.edu.pl (M.A.Z.); 2 Department of Nutritional Sciences, University of Vienna, 1090 Vienna, Austria; petra.rust@univie.ac.at (P.R.); dorota.majchrzak@univie.ac.at (D.M.)

**Keywords:** baby-led weaning (BLW), breastfeeding, complementary feeding, complementary foods, dietary patterns, infant feeding practices, maternal concerns, salt, sugar

## Abstract

Children aged <2 years should not be given meals with the addition of salt and sugar due to health risks and to promote healthier dietary habits. The aims of this study were: to assess the prevalence of the use of added salt (AS), sugar (ASu) and both salt and sugar (AS&Su) in the diets of Polish and Austrian toddlers aged 12–24 and 25–36 months; to explore the sociodemographic and early nutritional factors associated with the use of AS and ASu; to investigate the difference in dietary habits and maternal concerns about toddlers’ eating regarding the use of AS and ASu in toddlers’ diet. This cross-sectional anonymous study was conducted in 5893 mothers of children aged 12–36 months, recruited through social media in 2017–2019. The questionnaire consisted of questions about sociodemographics, early feeding practices and current children’s nutrition (e.g., use of AS and ASu, food frequency questionnaire). Multivariate logistic regression and cluster analyses were applied. Austrian mothers more often used AS than mothers from Poland (at 2 years old: 74.8% vs. 52.8%; at 3 years old 87.4% vs. 74.4%, *p* ≤ 0.001), however Polish mothers were more prone to use ASu (at 2 years old: 34.7% vs. 27.7%; at 3 years old: 59.0% vs. 45.8%, *p* ≤ 0.001). In younger toddlers (12–24 months), the odds of using of AS, ASu, and AS&Su increased with toddlers’ age, when the mother was a multipara, was not currently breastfeeding, or had exclusively breastfed for 4–5 months. This risk decreased when older toddlers (25–36 months) were introduced to solids by baby-led weaning (BLW). Toddlers from both countries who consumed meals with AS or ASu more often a followed Western-like dietary pattern. Our study emphasizes the need for parental nutritional education when beginning to introduce solid foods.

## 1. Introduction

The first 1000 days of children’s intensive growth and development are pivotal times during a life course. According to the Developmental Origins of Health and Disease hypothesis (DOHaD), environmental exposures during prenatal life, infancy and toddlerhood can modify the risk of many metabolic and non-communicable diseases in further life [1]. Moreover, this time is considered a sensitive period for establishing healthy dietary habits [2]. Breastfeeding, complementary feeding, parental feeding practices, but also social and family environment, play a crucial role in the development of children’s food preferences, attitudes, and dietary patterns [2,3,4]. However, children are evolutionarily predisposed to prefer high-energy, sweet and salty foods [2,5], as well as to reject bitter or new food items which may be extremely challenging for parents. Additionally, it was reported that repeated exposure to healthy foods (e.g., vegetables) along with limited access to unhealthy ones is a powerful factor that contributes to the development of healthier food preferences and dietary habits that may provide long term health outcomes [2,3,4]. Nonetheless, it emphasizes the need to offer children foods that are nutrient-dense and appropriate for them [2,4,6].

Health organizations and experts are in agreement that salt and sugar should be avoided during the first two years of life and limited later on [7,8,9,10]. Sodium and sugar intake in childhood was linked with many adverse health effects, including high blood pressure [11] and cardiovascular diseases [12], obesity [13,14] or dental caries [15]. Interestingly, in animal studies, it was reported that salt and sugar consumption may alter gut microbiota [16,17], which seems to play a pivotal role in health programming [18]. Another possible implication of early exposure to salt and sugar refers to the development of preference for salty and sweet tastes. It has been suggested that exposure to salty or sweet foods leads to an increase in acceptance and preference of saltiness and sweetness, but results are equivocal [5,6,19]. Moreover, adaptation to salty and sweet tastes during childhood may be transferred into adulthood, leading to higher sodium and free sugars intake throughout the lifespan [5,20]. Previous studies reported that sodium and added sugar intake increases with age by ~250 mg of salt/day per year from the age of 5 years [21] and from 2.3% of energy from added sugars in infancy to 13.4% in primary school pupils [22].

Previous studies that mainly assessed the intake of sodium, free or added sugars and use of added salt and sugar provide explicit results that the majority of young children may have exceeded sodium and free/added sugars recommendations since the earliest periods when they began eating solids [22,23,24,25,26,27,28]. As early introduction of those ingredients is a risk factor of many diet-related non-communicable diseases, which are a public health challenge worldwide [29,30], recognition of the factors associated with the use of salt and sugar is highly important. However, there is still little research examining concurrent use of added salt, sugar and both salt and sugar, especially according to various factors, such as sociodemographic or feeding-related. Hence, to fill the aforementioned scientific gaps, the aims of this study were: (1) to assess the prevalence of use of AS, ASu and AS&Su in the diets of Polish and Austrian toddlers aged 12–24 and 25–36 months; (2) to explore the sociodemographic and early nutritional factors associated with the use of AS, ASu and AS&Su in toddlers’ diets; (3) to investigate the difference in dietary habits and maternal concerns about toddlers’ eating regarding the use of AS, ASu and AS&Su in toddlers’ diets. The study was conducted in Poland and Austria, because of differences in complementary feeding practices in both countries as well as sociodemographic differences regarding income, maternity (parental) leave and family structure.

## 2. Materials and Methods

### 2.1. Study Design and Participants

This Internet-based cross-sectional study was conducted between 2017 and 2019 and investigated mothers of toddlers aged 12–36 months from Poland and Austria. The study design, methods and sample selection were described in detail in a previously published paper [31]. The study was performed in compliance with the Helsinki Declaration. As all data were collected anonymously and IP addresses were not collected following Polish and Austrian law, our study did not need ethical approval.

**Figure 1:**
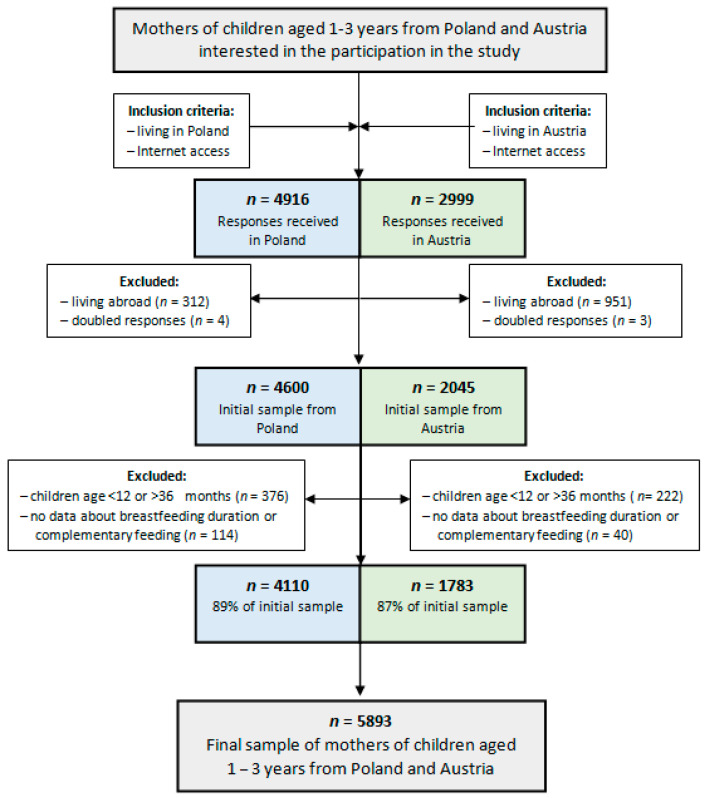
Flowchart of study sample collection.

Participants were recruited via social media. They received information about the anonymity of the study, the voluntariness and the possibility to stop their participation at any study stage. Questionnaires in both Polish and German were published using the online tool Google Forms and were completed by 7915 individuals, whereas the final analysis was conducted on 5893 subjects (Figure 1). Participants were excluded due to living abroad, not meeting inclusion criteria about children’s age, doubled responses, as well as missing any data.

Detailed study group characteristics from both countries according to age group are presented in Table 1. Most of the mothers from both countries were at least 30 years old, with an average or good economic situation. In most toddlers, solids were introduced between 4 and 6 months of age, using mixed-method and homemade complementary foods. In both countries, mothers of younger toddlers were more often 25–29 years old and primipara. Moreover, younger toddlers were more often currently being breastfed (*p* ≤ 0.001).

### 2.2. Questionnaire

#### 2.2.1. Demographic Data

Data about maternal and paternal age and education level, average monthly income per capita in the household, number of adults and children living in the household, as well as maternal marital status were collected. Moreover, information about the living area, both size of place of residence (recategorized as rural or urban), as well as the region of the country (which was further categorized according to the percentage of EU-28 average gross domestic product (GDP) per capita [32]) was gathered. Additionally, data about children’s gender, current age and data about birth outcomes were obtained. Detailed information about collected data was reported previously [31].

#### 2.2.2. Early Feeding Practices

Data about the duration of any breastfeeding (BF) and exclusive breastfeeding (EBF), as well as current breastfeeding were collected. Mothers were also asked about formula feeding, including information about giving formula to infants at the maternity ward. According to the World Health Organization (WHO), exclusive breastfeeding was defined as infant feeding with only breastmilk without introducing other foods, drinks or water (except medicines, vitamins or minerals) into the infant diet [33]; therefore, newborns who received formula at the maternity ward were categorized as non-exclusively breastfed. Data about complementary feeding, i.e., timing of introduction (CFI) and method (traditional spoon-feeding (TSF), baby-led weaning (BLW), and partially BLW) of introducing complementary foods were also collected. TSF was defined as solely or mostly spoon-fed by an adult, BLW was defined as baby feeding themselves, and mixed-method was defined as about half spoon-feeding by an adult and half baby feeding themselves. Mothers were also asked about types of complementary foods (CF) that were used during complementary feeding (commercial CF, homemade especially for infant or family foods).

#### 2.2.3. Toddlers’ Dietary Habits

Mothers were asked about toddlers’ feeding behavior during the last 3 months, including the use of AS and ASu. Throughout this paper, phrases “added salt” (AS) and “added sugar” (ASu) refer to the salt and sugar that the parents added during cooking or serving foods for their children. It is not tantamount to the term “added sugars”, which is defined by the European Food Safety Authority (EFSA) as “sucrose, fructose, glucose, starch hydrolysates (glucose syrup, high-fructose syrup) and other isolated sugar preparations used as such or added during food preparation and manufacturing”. Based on those questions, simultaneous use of AS and ASu was calculated (AS&Su). Information about frequency of consumption of 17 food items (raw vegetables; cooked vegetables; fruits; natural grain products; sweetened grain products; natural milk products; sweetened milk products; homemade meat dishes and products; processed meat; fish; eggs and egg dishes; legumes; commercial sweets, chocolates and cakes; homemade sweets, chocolates and cakes; fruit or fruit-vegetable juices; vegetable juices; water) was collected according to the following categories (1) never or almost never; (2) less than 1 time per week; (3) 1 time per week; (4) at least 2–4 times per week; (5) 1 time per day; (6) several times per day. Additionally, mothers were asked about methods of consuming meals by their children, their concerns about their toddlers’ eating behaviors and sources of knowledge about children’s feeding.

### 2.3. Statistical Analysis

All statistical analyses were conducted separately according to both (1) country and (2) age group: 12–24 months or 25–36 months. For nominal variables, results were presented as a percentage, and a chi-square test was performed. Factors associated with the use of AS, ASu and AS&Su were investigated using univariate and multivariate logistic regression analysis. The models included sociodemographic (maternal age and education, parity, living area, macroeconomic region and average monthly income per capita), and early feeding-related variables (CFI, complementary feeding method, types of CF, currently BF and duration of EBF).

Dietary patterns were determined using the *k*-means algorithm separately for both countries but without distinction of age group. Three clusters were selected because they were best interpretable: (1) pro-health, characterized by a high intake of vegetables, fruits, natural milk and grain products, fish, eggs and egg dishes, legumes, water, while low intake of sweetened milk and grain products, homemade and processed meats; (2) Western-like, characterized by a high intake of sweetened milk and grain products, homemade and processed meat, sweets, chocolates and cakes, juices, while low water intake; (3) non-eaters, characterized by lowest or one of the lowest intake of all of the food groups. All analyses were performed using the Statistica 13.3 software (TIBCO Software Inc., Palo Alto, CA, USA). For all analyses, *p* ≤ 0.05 was considered significant.

## 3. Results

### 3.1. Use of Added Salt, Sugar and Both Salt and Sugar

**Figure 2:**
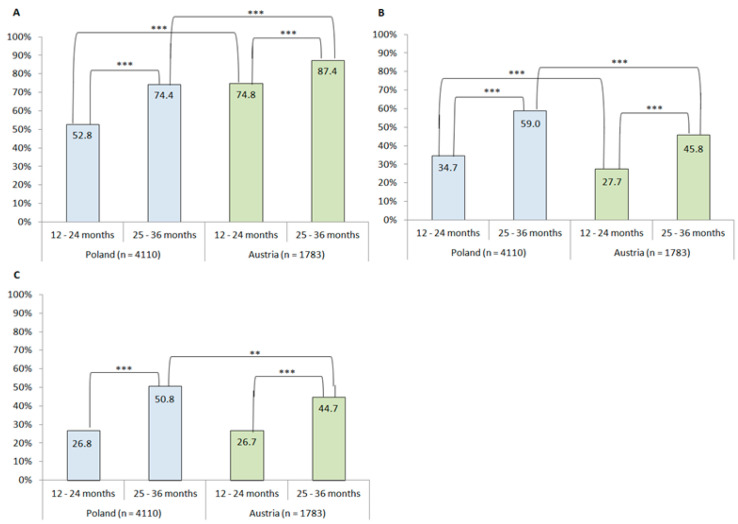
Use of added salt (**A**), sugar (**B**), both salt and sugar (**C**) in toddlers’ diets according to country and age group; ** *p* ≤ 0.01; *** *p* ≤ 0.001.

In total, AS, ASu and AS&Su were added into the diet of 58.5%, 32.9%, and 26.8% of toddlers aged 12–24 months (younger group) and 79.2%, 54.1%, and 48.5% aged 25–36 months (older group), respectively (results not presented). Austrian mothers tended to use AS more often than Polish, whereas mothers from Poland were more prone to use ASu and AS&Su (but only in the older group) more often (Figure 2).

### 3.2. Factors Associated with the Use of Added Salt, Sugar and Both Salt and Sugar in Toddlers’ Diets

#### 3.2.1. Sociodemographic Factors

The results of the multivariate logistic regression analysis conducted in the Polish sample adjusted to sociodemographic and early feeding-related factors are presented in Table 2. The odds of use of AS, ASu, and AS&Su increased with toddlers’ age, but only in the younger group. Factors increasing those odds were multiparty (AS, ASu, and AS&Su in both age groups), younger maternal age (ASu and AS&Su in the older group) and moderately high average monthly income (AS and AS&Su in the younger group). Those odds decreased when mothers were older (ASu and AS&Su in the younger group). In univariate models (Table S1), those odds increased in the group of mothers with lower education (AS and AS&Su in the younger group), living in a rural area (ASu, AS&Su in the older group), or mothers with a low monthly income (AS, ASu in the younger group).

Meanwhile, in Austria (Table 3), a multivariate logistic regression model showed that the odds of use of those ingredients increased with toddlers’ age (AS, ASu, and AS&Su in the younger and AS&Su in the older group). Other factors increasing those odds were maternal multiparity (only in the younger group) and living in a rural area (ASu and AS&Su in the younger group). Moreover, odds of use of ASu and AS&Su decreased when mothers were living in the macroeconomic region with the lowest or the highest GDP (older group), but low monthly income increased the odds of use AS (older group). In univariate models (Table S2), those odds increased when the family was living in a rural area (AS in the younger group), or with toddlers’ age (ASu in the older group).

#### 3.2.2. Early Feeding Factors

A multivariate logistic regression analysis conducted in the Polish sample adjusted to sociodemographic and early feeding-related factors (Table 2) showed that the odds of use of all ingredients were lower when CF were introduced using BLW method (except the use of ASu and AS&Su in the younger group). Additionally, in the younger group, those odds were higher when introducing CF using the TSF method (AS), when toddlers were never exclusively breastfed (ASu) or were exclusively breastfed for 4–5 months (AS, ASu, AS&Su). In the older group, the odds of the use of AS were higher when toddlers were not currently being breastfed and were never breastfed, exclusively breastfed or were exclusively breastfed for 4–5 months. The univariate models showed that the odds of the use of all ingredients increased in both groups when CFI occurred between 4 and 6 months (except ASu in the younger group). Moreover, in the younger group, those odds increased when mothers used commercial and homemade CF (AS) and toddlers were not currently being breastfed (AS, ASu, AS&Su), were never exclusively breastfed (AS&Su) or were exclusively breastfed for only 1–3 months (AS).

On the other hand, in Austria, the odds of use of AS, ASu and AS&Su in the younger group were higher when toddlers were not currently being breastfed (Table 3). In the younger group, EBF for 1–3 months decreased the odds of the use of AS. In the older group, the odds of the use of ASu and AS&Su were higher when toddlers were never breastfed. Additionally, lack of EBF and EBF for 4–5 months increased the odds of the use of ASu. Univariate models (Table S2) revealed that introducing CF by BLW method decreased the odds (AS in the younger group) and using commercial CF or CFI between 4 and 6 months increased those odds (AS in the older group).

### 3.3. Current Toddlers’ Dietary Habits, Maternal Concerns and Feeding Practices According to the Use of Added Salt, Sugar and Both Salt and Sugar

Polish toddlers in both age groups who had received meals with AS, ASu, and AS&Su more often followed a Western-like and less pro-health dietary pattern. Moreover, in those groups, more mothers reported concerns about toddlers’ eating and poorer feeding practices were observed (Table 4). Meanwhile, in the Austrian sample, similar differences in dietary habits were observed only with the use of ASu and AS&Su in both age groups (Table 5).

### 3.4. Sources of Knowledge about Children’s Feeding According to the Use of Added Salt, Sugar and Both Salt and Sugar

Furthermore, in the present study, associations between sources of knowledge about children’s feeding and the use of AS, ASu, and AS&Su were evaluated (Table S3). Mothers who used those ingredients tended to report more often family and friends (in Austria, only in relation to ASu and AS&Su in the younger group), but less often nutritionists or dietitians as a source of knowledge acquisition (Poland: AS and AS&Su in the younger group; Austria: AS, ASu, AS&Su in the younger and AS in the older group). Moreover, Polish mothers more often indicated magazines (AS in the younger group), TV (AS, ASu, and AS&Su in the younger group), but less often books, as a source of knowledge acquisition (AS and AS&Su in the younger group; AS, ASu, and AS&Su in the older group). Additionally, in the Austrian sample, those mothers who used ASu and AS&Su more often declared doctors or midwives as their source of knowledge about children’s feeding.

## 4. Discussion

The present study demonstrated a high prevalence of the use of added salt, and lower of added sugar and both ingredients in the diets of the Polish and Austrian toddlers. Furthermore, those rates were higher in toddlers aged 25–36 months compared to 12–24 months. It was also observed that the strongest sociodemographic factor associated with the use of added salt, sugar and both salt and sugar was multiparity. Among early feeding factors, current breastfeeding, duration of exclusive breastfeeding and method of introducing complementary foods were the strongest. Moreover, toddlers who consumed meals with added salt and sugar also showed poorer dietary patterns and meal environment. Interestingly, it was also observed that those mothers who used added salt (and sugar to a lesser extent) tended to be more concerned about their children’s feeding practices. The obtained results were specific for the country, as well as diminished with toddlers’ age.

### 4.1. Prevalence of the Use of Added Salt and Sugar in the Toddlers’ Diets

Half of the Polish mothers and three-quarters of the Austrian mothers declared the use of added salt in the diet of their two year old toddlers; for added sugar, these came to one-third for the Polish mothers and one-quarter for the Austrian mothers. Therefore, those rates were higher among older toddlers. Previous studies reported that from 25% (France [27]) to 50% (USA [34]) and 60% (Sri Lanka [35]) of infants received meals with the addition of salt before the end of 12 months of age. A study from Australia showed that around 40% of toddlers aged 2–3 years consumed meals with added salt, and 11% of them added salt at the table [24]. Sugar was added into the diet of 25% of 10 month infants from France [27] and 42% of infants from Sri Lanka [35]. These rates increased with children’s age, which is in accordance with the results of other studies conducted in Europe [27,36,37] and was also confirmed in the present study. The odds of the use of added salt and sugar into toddlers’ diets increased by 18% and 14% (Poland) and 16% and 13% (Austria) per month in the second year of life, respectively. Whereas in the present study, only the use of added salt and sugar as ingredients was assessed, various studies investigated the use of added salt or sugar as well as the intake of salt or sodium and added or free sugars. The use of added salt and sugar intake is inseparably associated with sodium and added/free sugars intake, which also increased with children’s age [21,22,23,28,37,38,39,40,41]. Unfortunately, many infants and toddlers around the world have intakes of sodium [25,26,38,39,42,43] and added/free sugars [22,28,37,39,40,43,44,45] already close to the upper limit or even above recommendations. Interestingly, many studies conducted on infants and children have shown that boys compared to girls had a higher intake of sodium [21,23,26,46]. Concerning sugar intake, the results are inconclusive. Some studies demonstrated higher intake among girls than boys [40], whereas in the study conducted by Yuan et al. [47], boys were more prone to consume added sugar, however, it was only observed at 8 months of age. Nonetheless, in our study, we did not observe any gender differences in the prevalence of use of added salt, sugar and both salt and sugar (results not presented).

### 4.2. Sociodemographic Factors Associated with the Use of Added Salt and Sugar in the Toddlers’ Diets

Multiparity was associated with the use of added salt and sugar in the toddlers’ diets, whereas maternal age, education, and the economic situation showed limited influence. Multiparity was the strongest factor that increased the odds of use of added salt, sugar and both ingredients by 32–85% in the diets of the Polish toddlers aged 12–36 months and the Austrian toddlers aged 12–24 months. Previously, it was reported that Japanese 3 year old toddlers with older siblings exhibited a higher urinary sodium excretion, as well as daily consumption of snacks compared to firstborn children [48]. Results concerning the use of added sugar and parity are ambiguous, as in the study of Marinho et al. [40], no association between having siblings and use of added sugar was found, but Yuan et al. [47] demonstrated that consumption of added sugar was associated with having a multiparous mother. Our results are consistent with previous results from France [27] and observations from a focus study conducted in five European countries, in which some parents declared following the nutritional guidelines for the firstborn child, but for the second one they “did not follow them as closely” and in consequence, dietary practices were poorer [49,50]. Maternal education remained not significant after adjustment for covariates. Whereas poorer early feeding practices were associated with maternal lower education level [31,51,52], no associations regarding salt use or sodium intake in infants and toddlers [25,26,34] were observed, in contrast to free sugars intake in children [40]. Moreover, a limited inverse association between economic situation and use of added salt and both ingredients in the diet of the Polish toddlers aged 12–24 months, as well as salt in the diet of the Austrian toddlers in this age was noticed. Although a lower economic situation was previously linked to poorer feeding practices during the first years of life [53], it was not observed in relation to sodium [46] or added sugars intake [37], nor sugar and salt use [27], as well as the timing of introducing solids analyzed in our previous study [31].

### 4.3. Early Feeding Factors Associated with the Use of Added Salt and Sugar in the Toddlers’ Diet

A lack of current breastfeeding increased the odds of use of added salt, sugar, and both of them in the younger group from Austria, as well as the odds of the use of added salt in the older group from Poland. Additionally, a lack or lower duration of exclusive breastfeeding increased the odds of use of added sugar and both salt and sugar in the younger group, and use of added salt in the older group from Poland, as well as the use of added sugar and both ingredients in the older group from Austria. These results are in agreement with previous studies that linked breastfeeding with better dietary habits during infancy and subsequent life [23,42,54] and lower sodium intake in breastfed children [25,26,42]. Nevertheless, in the study of Yuan et al. [47], intake of added sugars was not significantly associated with breastfeeding duration. Breastfeeding is also associated with higher intake of unprocessed foods and lower intake of processed foods, which are main sources of salt/sodium and added sugars in the diet [23,24,54,55]. Breastmilk is an important source of chemosensory information and plays a pivotal role in developing children’s food preferences [2,8]. Moreover, breastfeeding mothers tend to be older and better educated than those who are not breastfeeding their children [42,54]. This fact may lead to better compliance with nutritional guidelines and in consequence, healthier dietary and lifestyle habits in family and children [2,4,26,51,56,57]. In contrary to previous results, a study from France found that any breastfeeding duration was positively associated with use of added salt and sugar in infants’ diet [27]. The authors hypothesized that it may be caused by a choice of introducing homemade complementary foods rather than commercial ones, which was higher in breastfeeding mothers and was confirmed in other studies [27,54]. Unfortunately, several studies reported that homemade or family foods were often prepared with salt or sugar addition against nutritional recommendations and consequently, contributed into sodium or added sugars intake [8,10,25,26].

In the present study, we did not observe a significant association between the use of added salt or sugar and types of complementary foods. Interestingly, the use of commercial complementary foods tends to be a risk factor of the use of added salt in the younger Polish and older Austrian toddlers. Despite a lack of association in the present study, a previous longitudinal investigation reported that higher consumption of commercial baby foods increased the intake of added sugars in later childhood [22]. Analysis of commercial baby foods from four cities in the European region revealed that many of them contained more than the recommended amount of sodium and sugars, possibly due to the addition of salty or sweet ingredients, such as ham, cheese, or concentrated fruit juices [10]. These results are consistent with previous studies from the USA, which showed that 58% and 45% [58] or 84% and 70% [59] of commercial toddler foods had high sodium or added sugar levels, respectively. The authors emphasized that many commercial infant and toddler foods did not fulfill the criteria of healthy foods in accordance with age, despite parents’ perception [49,58].

Timing and method of introducing complementary foods into an infant’s diet are other aspects that might determine subsequent dietary habits and food preferences [2,8]. In a few previous studies, it was observed that too early starting of complementary feeding (before 4 months) was related to salt/sodium intake [25,26,27] and use of added sugar [27]. However, our study did not replicate these findings, as the association between the use of added salt, sugar, and age of introducing complementary foods did not remain significant after adjustment to maternal sociodemographic and early feeding factors. Moreover, early introducing of solids seems to be a plausible risk factor of the use of added salt in toddlers’ diet. Parents who introduced solids earlier had more time to introduce salt and sugar [27], and those children were characterized by poorer dietary patterns or feeding difficulties [56,60]. This may be partially explained by poorer following of nutritional recommendations [57] and maternal characteristics [31,51,53], similar to breastfeeding.

Finally, the method of introducing complementary foods may be an important factor that contributes to shaping dietary habits and food preferences [61]. We observed that following the BLW method decreased odds of use of added salt in the diet of the Polish toddlers, whereas traditional spoon-feeding increased them in the younger group. Moreover, BLW decreased odds of use of added sugar and both ingredients in the older toddlers. Previous studies have shown equivocal results of sodium intake according to the method of introducing solids. Morison et al. [62] reported no difference in intake of sodium or sugars according to the method of introducing solids, whereas in a study of Erickson et al. [38], infants who were introduced to solids using a modified BLW method (BLISS) had a higher intake of sodium at 7 months, but not at 24 months, with no difference in added sugars intake. Those differences may be caused by higher consumption of family foods than commercial complementary foods during feeding by the BLW method [63]. Our results may be explained in two ways. First, infants who were introduced to solids by the BLW method tend to be breastfed longer, introduced to complementary foods later, and their mothers had better characteristics which lead to healthier feeding practices [31,62,63,64,65,66]. Second, the BLW method is associated with more responsive parental feeding practices [64] and was linked to lower fussiness in later childhood [65].

### 4.4. The Use of Added Salt and Sugar and Toddlers’ Dietary Habits and Maternal Concerns About Eating

Interestingly, in the present study, toddlers’ dietary patterns varied under consideration of the consumption of meals with or without added salt or sugar. The Polish and Austrian toddlers who consumed added salt and sugar more often followed a Western-like pattern and less often pro-health pattern, characterized by more frequent consumption of low-processed foods, such as vegetables and fruits, grains, or water. Those results are consistent with previous studies that linked salt intake with consumption of sugar-sweetened beverages [67] or consumption of savory snacks with sugar-sweetened beverages and sweets [56]. Toddlers who a followed Western-like dietary pattern may be a risk group of higher sodium/salt [24,26,39,68,69] or sugars [37,39,40,45] intake from processed foods which are characteristic of this pattern. Moreover, higher consumption of salty and sweet foods may blunt the appetite and decrease the intake of nutrient-dense food [35]. Furthermore, early exposure to unhealthy foods is associated with enhanced preference and increased intake of these foods in later childhood [23,70]. In the present study, around one-quarter of the younger and half of the older toddlers from both age groups consumed meals with added both salt and sugar. The results are in accordance with previous studies which reported that the introduction of those ingredients into infants’ or toddlers’ diets is highly associated with each other [23,27,34,35,71]. This may be explained in several ways. Firstly, salty and sweet preferences in children are correlated with each other and are related to dietary intake of salty, but not sweet foods [72]. Secondly, a lot of commercial complementary foods contain not only too much salt/sodium, but also sugar [58,59]. Thirdly, mothers who do not follow strictly nutritional guidelines about salt may be more likely to also add sugar during the complementary feeding period [57]. Furthermore, Polish toddlers, who consumed meals with added salt and sugar, were more often eating meals during watching TV, which may be one of the predictors of poorer dietary habits [56].

Regarding the results considering the mothers’ concerns about their toddlers’ eating behaviors, it could be noticed that, the Polish mothers who prepared meals without the addition of salt and sugar were less concerned about their toddlers’ eating, whereas in other groups, concerns about “eating too little” (only salt and both ingredients), “not eating vegetables” or “not eating novel foods” were reported more often. Therefore, we hypothesized that those mothers may use added salt and sugar as an encouraging strategy to increase the intake of those foods, as mothers who are more concerned about children’s eating are prone to have less responsive feeding practices [4]. Furthermore, the addition of salt, but not sugar, has a dose-dependent influence on the toddlers’ vegetable intake [73]. However, in a previous study from France, the authors did not observe any association between maternal concerns about the child’s health and salt or sugar use during infancy [27].

### 4.5. Sources of Knowledge About Children’s Feeding

Presented results showed that mothers of the Polish toddlers who used added salt and sugar in children’s meals more often declared family or friends and TV as sources of knowledge about children’s feeding, whereas nutritionists or dieticians were reported less often. Similar results were observed previously by Bournez et al. [27] in regard to sugar, but not salt use. However, in both studies, the Internet was the main source of knowledge about nutrition. Previously, relatively high parental consciousness about health risks and the necessity of reduction in salt and sugar in children’s feeding were reported [48,71]. Morinaga et al. [48] did not find any associations with salt intake in children, whereas Khokhar et al. [71] reported that those parents more often declared behaviors aimed to reduce salt intake in children. Interestingly, some studies suggest that children of parents who restricted sugar intake have increased sweetness preference [5]. Those results emphasize the necessity of educational interventions to improve parental practices from the beginning of the introduction of solids into the infant’s diet.

Most of the results observed in the present study were significant only in the Polish sample, especially the younger group. A potential explanation for this difference between results obtained in both evaluated countries is the higher prevalence of salt addition in Austrian toddlers. Nearly 90% of older Austrian toddlers received meals with added salt, and this fact could lead to diminishing gaps in toddlers’ characteristics within both subgroups. Moreover, the strength of the factors associated with the use of added salt may also reduce because of the use of added salt increases with toddlers’ age. In our previous paper, which was a part of the present study, we also observed that the Austrian mothers were following the WHO recommendations about the age of introduction of complementary feeding less often than the Polish mothers [31].

### 4.6. Strengths and Limitations

An important strength of this study was the large sample size (*n =* 5893) from two European countries. Due to the study design and administration of the questionnaire via the Internet, it was possible to reach out to mothers from diverse parts of Poland and Austria and provide equal access for participation over geographical and financial barriers. Moreover, we assessed the prevalence of the use of added salt, sugar, and both of them in two age categories, which is important because of the fact that children younger than 2 years should not be given added salt nor sugar [10]. Further, our study was able to explore some plausible factors associated with the use of added salt and sugar, both sociodemographic and early feeding, as well as to investigate the association between dietary habits among children with different statuses of use of added salt and sugar. It is worth emphasizing that some of the presented factors may be modifiable. This creates the possibility of interventions aimed to improve early dietary habits and even reduce the risk of non-communicable diseases in further life.

Notwithstanding, the present study has some limitations, which should be considered. First of all, the survey was conducted only among Internet users. However, Internet access is generally available in Poland, as well as in Austria. In Poland, 99% of households with children have Internet access, and in Austria, 88% [31,74]. Second, all of the data about early feeding factors were self-reported by mothers of toddlers aged 12–36 months and they could have some difficulties in remembering details. Although, existing data suggest that maternal recall is valid years later, whereas information about introducing solids may be poorer reported [75,76,77,78]; nonetheless, the recall time in the present study was relatively short (≤3 years), therefore, recall bias was minimized. Third, we collected data only about the current use of added salt and sugar. No information about the intake of salt/sodium, free/added sugars, or products that contain high amounts of salt or sugar, as well as the time when salt and sugar were introduced into children’s diet is available. Fourth, there is a likelihood that the present investigation involved more mothers who were especially interested in children’s nutrition, thereupon they could follow the nutritional recommendations to a higher degree. Nonetheless, the present study demonstrated a low adherence to the recommendation of avoiding salt and sugar during complementary feeding, as we observed a high prevalence of use of added salt (especially in the Austrian toddlers) and moderately high prevalence of use of added sugar. Moreover, we found some interesting influencing factors (e.g., related to early feeding practices or maternal concerns about children eating behaviors), which can be directions to develop intervention strategies and educational programs.

## 5. Conclusions

The most worrisome finding from the present study is that at least half of the Polish and majority of the Austrian mothers, especially of the older toddlers, did not follow the recommendations about avoiding salt use during the first years of children’s life. Even though the prevalence of the use of added sugar was lower, those results are also disconcerting, especially as the use of sugar, like salt, should be limited in children’s nutrition. Furthermore, the obtained results revealed that the use of added salt, sugar, or both salt and sugar in the toddlers’ diet was associated with multiparity and lower maternal age. Besides, we found that shorter duration of exclusive breastfeeding and lack of any or current breastfeeding, as well as traditional spoon-feeding, may increase the risk of use of added salt, sugar, or both of them, whereas introducing solids using the BLW method may decrease it. Especially alarming is that non-compliance with salt and sugar recommendations was related to other unhealthy feeding practices and poorer dietary habits (Western-like vs. pro-health dietary patterns) during infancy and toddlerhood.

As higher salt/sugar intake in childhood potentially leads to becoming accustomed to high levels of saltiness/sweetness in food, this in turn, aids improper food choice later in life. Taking this into account, as well as possible implications for long-term health effects that arise from exceeding salt and sugar consumption, some efforts should be taken to lower intake of those ingredients. Those actions should include creating political, social and food environment that supports the development of healthy dietary habits associated with low intake of added salt and sugar. Our results indicate that those interventions to promote a healthy diet should start very early, ideally at the beginning of introducing complementary foods.

## Figures and Tables

**Table 1:** Study group characteristics according to sociodemographic factors.

Variables	Poland (*n* = 4110)%			Austria (*n* = 1783)%		
	12–24 Months (*n* = 2680)	25–36 Months (*n* = 1430)	*p*-Value	12–24 Months (*n* = 941)	25–36 Months (*n* = 842)	*p*-Value
Maternal age:			≤0.001			≤0.001
<25 years	5.3	3.4		6.4	2.7	
25–29 years	36.5	24.8		28.4	23.3	
30–34 years	44.7	53.4		39.4	40.1	
35–39 years	12.2	16.4		22.4	27.2	
≥40 years	1.3	2		3.4	6.7	
Maternal education:			0.294			0.557
Primary and vocational	1.1	0.7		27.2	25.1	
High school	12	11		3.4	3.8	
University	86.9	88.3		69.4	71.1	
Household size:			≤0.001			≤0.001
2 persons	2	2		1.5	3	
3 persons	66.8	52.1		55.5	36.2	
4 persons	23.6	38.3		30.9	46.3	
5 persons	5.5	5.7		8.5	10.1	
≥6 persons	2.1	1.9		3.6	4.4	
Parity:			≤0.001			≤0.001
Primiparas	73.3	55.8		57.1	38.2	
Multiparas	26.7	44.2		42.9	61.8	
Living area:			0.601			0.839
Rural	17.5	16.9		56.5	56.1	
Urban	82.5	83.1		43.5	43.9	
Living macroeconomic region (GDP EU-28 average):			0.339			0.495
47–50%	14.4	12.7		-	-	
51–100%	62.3	63.2		5.7	5.3	
101–110%	23.3	24.1		43.6	40.3	
111–130%	-	-		25.8	26.7	
131–150%	-	-		9.3	11.3	
>150%	-	-		15.6	16.4	
Average monthly income per capita ^1^:			0.468			0.008
1st category	1.5	1.5		8.3	8.7	
2nd category	15	13.5		20.6	21	
3rd category	32.7	32.8		33.5	29.3	
4th category	17.7	16.6		27.5	25.1	
5th category	11.6	13.3		8.5	12.9	
6th category	21.5	22.3		1.6	3	
Infant gender:			0.628			0.211
Girl	48.1	47.3		51.5	48.6	
Boy	51.9	52.7		48.5	51.4	
Timing of CFI:			≤0.001			0.878
<4 months	2	3.4		4.3	4.5	
4–6 months	59.4	62.6		74.9	75.5	
≥7 months	38.6	34		20.8	20	
Complementary feeding method:			0.004			0.076
TSF	26.9	30.2		38.9	33.7	
mixed	56.6	51.1		47.7	51.4	
BLW	16.5	18.7		13.4	14.9	
Types of CF:			0.228			0.733
Ready-to-eat foods	22	21.7		22.7	24	
Homemade foods	48.5	46.2		51.5	51.4	
Family foods	29.5	32		25.8	24.6	
Currently BF:			≤0.001			≤0.001
No	38.8	72		62.4	81	
Yes	61.2	28		37.6	19	
EBF duration:			≤0.001			0.506
Never BF	5.3	5.5		8.1	9.5	
Never EBF	50	51.4		32.1	28.3	
1–3 months	1.9	2.4		6.3	7.5	
4–5 months	7.8	11.3		25.5	25.7	
6–7 months	34.7	28.6		25.5	26.7	
>7 months	0.3	0.8		2.5	2.6	

^1.^ Average monthly income per capita categories depends on country: 1st category < 500 PLN (Poland)/< 1000 EUR (Austria); 2nd category 500–1000 PLN/1000–1500 EUR; 3rd category 1001–2000 PLN/1501–2000 EUR; 4th category 2001–2500 PLN/2001–3000 EUR; 5th category 2501–3000 PLN/3001–5000 EUR; 6th category ≥3001 PLN/≥5001 EUR; BF—breastfeeding; BLW—baby-led weaning; CF—complementary foods; CFI—complementary feeding introduction; EBF—exclusive breastfeeding, TSF—traditional spoon-feeding.

**Table 2:** Multivariate regression analysis models of factors influencing the use of added salt, sugar, and salt and sugar in the Polish toddlers (*n* = 4110).

Factors	Variable	12–24 Months			25–36 Months		
		(*n* = 2680)			(*n* = 1430)		
		AS aOR (95% CI)	ASu aOR (95% CI)	AS&Su aOR (95% CI)	AS aOR (95% CI)	ASu aOR (95% CI)	AS&Su aOR (95% CI)
**Sociodemographic Factors**	Toddler age:	1.18 (1.15–1.21) ***	1.14 (1.11–1.17) ***	1.16 (1.13–1.19) ***	1.01 (0.98–1.05)	1.02 (0.99–1.05)	1.01 (0.98–1.04)
	Maternal age:						
	< 25 years	0.91 (0.61–1.38)	1.34 (0.89–2.02)	1.09 (0.70–1.70)	1.07 (0.48–2.38)	2.72 (1.31–5.65) **	2.22 (1.12–4.37) *
	25–29 years	1.01 (0.84–1.22)	1.11 (0.91–1.34)	1.11 (0.91–1.37)	1.23 (0.90–1.69)	1.49 (1.13–1.96) **	1.43 (1.09–1.87) **
	30–34 years	1	1	1	1	1	1
	35–39 years	0.98 (0.75–1.28)	0.79 (0.60–1.05)	0.80 (0.59–1.08)	1.36 (0.95–1.94)	0.92 (0.68–1.25)	1.11 (0.82–1.50)
	≥ 40 years	0.59 (0.28–1.23)	0.20 (0.07–0.59) **	0.27 (0.09–0.83) *	1.54 (0.60–3.94)	0.71 (0.33–1.52)	0.88 (0.41–1.92)
	Maternal education:						
	Primary and vocational	2.03 (0.83–4.96)	1.39 (0.63–3.05)	2.11 (0.94–4.72)	2.29 (0.28–19.06)	1.15 (0.27–4.85)	1.65 (0.40–6.84)
	High school	1.08 (0.83–1.42)	1.04 (0.79–1.36)	1.18 (0.88–1.57)	1.65 (1.03–2.64) *	0.86 (0.59–1.26)	1.00 (0.69–1.45)
	University	1	1	1	1	1	1
	Parity:						
	Primiparas	1	1	1	1	1	1
	Multiparas	1.80 (1.47–2.20) ***	1.37 (1.12–1.67) **	1.55 (1.26–1.92) ***	1.32 (1.01–1.71) *	1.37 (1.09–1.72) **	1.38 (1.10–1.73) **
	Living area:						
	Rural	1.02 (0.82–1.27)	1.12 (0.90–1.40)	1.10 (0.87–1.39)	1.08 (0.76–1.52)	1.17 (0.87–1.58)	1.15 (0.86–1.54)
	Urban	1	1	1	1	1	1
	Macroeconomic region (GDP EU-28 average):						
	47–50%	1.07 (0.81–1.42)	0.83 (0.62–1.11)	0.79 (0.57–1.07)	0.86 (0.55–1.34)	1.05 (0.71–1.55)	0.95 (0.65–1.39)
	51–100%	0.99 (0.80–1.21)	0.93 (0.76–1.15)	0.86 (0.69–1.07)	0.98 (0.73–1.33)	1.13 (0.87–1.47)	1.11 (0.86–1.44)
	101–110%	1	1	1	1	1	1
	Average monthly income per capita (PLN):						
	<500	1.35 (0.64–2.84)	0.94 (0.45–2.00)	1.55 (0.71–3.37)	2.23 (0.61–8.08)	1.67 (0.57–4.86)	2.02 (0.74–5.54)
	500–1000	1.26 (0.90–1.75)	1.30 (0.92–1.82)	1.62 (1.12–2.36) *	1.16 (0.71–1.89)	0.85 (0.55–1.32)	0.94 (0.61–1.44)
	1001–2000	1.13 (0.85–1.49)	1.15 (0.86–1.53)	1.30 (0.94–1.80)	1.17 (0.79–1.73)	0.83 (0.58–1.19)	0.94 (0.66–1.34)
	2001–2500	1.40 (1.03–1.90) *	1.36 (0.99–1.86)	1.71 (1.21–2.42) **	1.32 (0.84–2.06)	1.01 (0.67–1.51)	1.02 (0.69–1.51)
	2501–3000	1	1	1	1	1	1
	≥3001	0.93 (0.69–1.25)	0.94 (0.69–1.28)	1.09 (0.77–1.55)	1.20 (0.79–1.81)	0.88 (0.61–1.28)	0.87 (0.60–1.26)
**Feeding-Related Factors**	Timing of CFI:						
	<4 months	1.24 (0.51–1.74)	1.01 (0.55–1.85)	0.82 (0.42–1.59)	1.31 (0.57–2.97)	0.69 (0.36–1.33)	0.80 (0.41–1.54)
	4–6 months	1.09 (0.91–1.32)	0.94 (0.78–1.14)	1.04 (0.84–1.28)	1.14 (0.86–1.52)	1.10 (0.85–1.43)	1.20 (0.93–1.54)
	≥7 months	1	1	1	1	1	1
	Complementary feeding method:						
	TSF	1.28 (1.04–1.58) *	0.95 (0.77–1.17)	1.03 (0.82–1.30)	1.06 (0.77–1.45)	1.09 (0.83–1.44)	1.06 (0.81–1.39)
	Mixed	1	1	1	1	1	1
	BLW	0.70 (0.55–0.90) **	0.95 (0.74–1.22)	0.81 (0.61–1.08)	0.69 (0.49–0.98) *	0.61 (0.44–0.83) **	0.63 (0.46–0.87) **
	Types of CF:						
	Commercial	1.18 (0.90–1.54)	0.79 (0.60–1.03)	0.88 (0.66–1.19)	0.60 (0.40–1.34)	0.74 (0.52–1.06)	0.76 (0.53–1.08)
	Homemade	1.01 (0.82–1.25)	0.93 (0.75–1.16)	1.04 (0.82–1.32)	0.73 (0.53–1.01)	0.80 (0.60–1.06)	0.78 (0.59–1.03)
	Family	1	1	1	1	1	1
	Currently BF:						
	No	1.08 (0.89–1.30)	1.02 (0.84–1.24)	1.02 (0.83–1.26)	1.71 (1.27–2.29) ***	0.96 (0.73–1.27)	1.15 (0.88–1.51)
	Yes	1	1	1	1	1	1
	EBF duration:						
	Never BF	1.06 (0.70–1.60)	1.65 (1.10–2.48) *	1.46 (0.94–2.27)	3.50 (1.69–7.27) ***	1.16 (0.65–2.09)	1.47 (0.83–2.60)
	Never EBF	1.04 (0.87–1.26)	1.11 (0.91–1.34)	1.12 (0.91–1.38)	1.34 (1.01–1.79) *	1.16 (0.90–1.51)	1.20 (0.92–1.55)
	1–3 months	1.77 (0.93–3.36)	0.92 (0.48–1.76)	1.28 (0.67–2.48)	1.35 (0.56–3.26)	1.05 (0.49–2.23)	1.33 (0.63–2.81)
	4–5 months	1.63 (1.15–2.32) **	1.86 (1.33–2.60) ***	1.82 (1.28–2.59) ***	1.65 (1.01–2.69) *	1.01 (0.67–1.52)	1.10 (0.74–1.65)
	6–7 months	1	1	1	1	1	1
	>7 months	0.61 (0.14–2.69)	0.17 (0.02–1.46)	0.26 (0.03–2.28)	0.77 (0.22–2.76)	0.98 (0.29–3.34)	1.02 (0.30–3.46)

aOR—adjusted odds ratio; AS—added salt; ASu—added sugar; AS&Su—added salt and sugar; BF—breastfeeding; BLW—baby-led weaning; CF—complementary foods; CFI—complementary feeding introduction; CI—confidence interval; CF—complementary foods; EBF—exclusive breastfeeding; GDP—Gross Domestic Product; TSF—traditional spoon-feeding; * *p* ≤ 0.05; ** *p* ≤ 0.01; *** *p* ≤ 0.001.

**Table 3:** Multivariate regression analysis models of factors influencing the use of added salt, sugar, and salt and sugar in the Austrian toddlers (*n* = 1783).

Factors	Variable	12–24 Months (*n* = 941)			25–36 Months (*n* = 842)		
		AS aOR (95% CI)	ASu aOR (95% CI)	AS&Su aOR (95% CI)	AS aOR (95% CI)	ASu aOR (95% CI)	AS&Su aOR (95% CI)
**Sociodemographic Factors**	Toddler age:	1.16 (1.11–1.21) ***	1.13 (1.08–1.18) ***	1.13 (1.08–1.18) ***	1.03 (0.97–1.09)	1.04 (1.00–1.08)	1.04 (1.00–1.08) *
	Maternal age:						
	<25 years	1.67 (0.78–3.57)	1.23 (0.64–2.37)	1.27 (0.66–2.46)	0.55 (0.16–1.82)	0.68 (0.28–1.69)	0.62 (0.24–1.55)
	25–29 years	1.15 (0.78–1.71)	1.11 (0.76–1.62)	1.08 (0.74–1.58)	0.73 (0.42–1.28)	0.80 (0.55–1.15)	0.82 (0.57–1.19)
	30–34 years	1	1	1	1	1	1
	35–39 years	0.96 (0.63–1.46)	0.88 (0.58–1.33)	0.89 (0.58–1.36)	0.89 (0.52–1.54)	0.97 (0.68–1.38)	0.94 (0.66–1.34)
	≥40 years	0.77 (0.32–1.85)	0.85 (0.36–2.01)	0.92 (0.39–2.19)	0.64 (0.28–1.50)	1.20 (0.66–2.16)	1.15 (0.64–2.08)
	Maternal education:						
	Primary and vocational	1.07 (0.73–1.59)	1.08 (0.76–1.54)	1.11 (0.77–1.59)	0.85 (0.50–1.43)	1.08 (0.76–1.53)	1.03 (0.73–1.47)
	High school	0.43 (0.19–1.05)	0.65 (0.26–1.61)	0.56 (0.22–1.46)	1.58 (0.34–7.28)	1.56 (0.73–3.35)	1.44 (0.67–3.08)
	University	1	1	1	1	1	1
	Parity:						
	Primiparias	1	1	1	1	1	1
	Multiparias	1.61 (1.14–2.28) **	1.84 (1.34–2.54) ***	1.85 (1.34–2.55) ***	0.82 (0.52–1.30)	1.09 (0.80–1.47)	1.11 (0.82–1.50)
	**Living area:**						
	Rural	1.23 (0.85–1.78)	1.57 (1.09–2.27)*	1.54 (1.06–2.24)*	0.96 (0.58–1.59)	1.13 (0.81–1.58)	1.08 (0.77–1.51)
	Urban	1	1	1	1	1	1
	Macroeconomic region (GDP EU-28 average):						
	47–50%	-	-	-	-	-	-
	51–100%	1.17 (0.55–2.48)	1.43 (0.75–2.73)	1.52 (0.79–2.91)	3.97 (0.89–17.66)	0.36 (0.18–0.72) **	0.38 (0.19–0.76) **
	101–110%	1	1	1	1	1	1
	111–130%	0.85 (0.58–1.26)	1.35 (0.93–1.96)	1.31 (0.89–1.91)	1.16 (0.69–1.94)	0.81 (0.57–1.15)	0.78 (0.55–1.10)
	131–150%	1.16 (0.64–2.12)	0.69 (0.37–1.27)	0.67 (0.36–1.25)	2.06 (0.86–4.94)	0.63 (0.38–1.02)	0.62 (0.38–1.01)
	>150%	0.82 (0.50–1.34)	1.31 (0.78–2.21)	1.25 (0.74–2.11)	0.95 (0.49–1.85)	0.61 (0.38–0.98) *	0.58 (0.36–0.93) *
	Average monthly income per capita (EUR):						
	<1000	0.80 (0.37–1.70)	1.00 (0.46–2.17)	1.27 (0.57–2.82)	1.53 (0.65–3.62)	0.95 (0.51–1.78)	0.89 (0.47–1.66)
	1000–1500	1.05 (0.55–2.02)	1.56 (0.82–2.97)	1.78 (0.91–3.48)	2.45 (1.16–5.18) *	1.22 (0.74–2.02)	1.21 (0.73–2.01)
	1501–2000	1.15 (0.63–2.10)	0.99 (0.54–1.82)	1.26 (0.66–2.38)	1.55 (0.81–2.97)	1.14 (0.71–1.83)	1.15 (0.71–1.85)
	2001–3000	1.04 (0.56–1.92)	1.21 (0.65–2.24)	1.46 (0.76–2.78)	1.60 (0.82–3.15)	1.10 (0.68–1.80)	1.10 (0.68–1.79)
	3001–5000	1	1	1	1	1	1
	≥5001	0.84 (0.22–3.17)	1.59 (0.44–5.75)	1.95 (0.53–7.10)	1.58 (0.41–6.06)	1.41 (0.57–3.47)	1.22 (0.50–3.01)
**Feeding-Related Factors**	Timing of CFI:						
	<4 months	1.04 (0.42–2.55)	0.57 (0.24–1.36)	0.55 (0.22–1.34)	1.75 (0.51–5.97)	0.93 (0.43–2.02)	0.80 (0.37–1.75)
	4–6 months	1.14 (0.74–1.76)	0.91 (0.59–1.40)	0.91 (0.58–1.40)	1.66 (0.93–2.96)	0.90 (0.60–1.35)	0.89 (0.59–1.34)
	≥7 months	1	1	1	1	1	1
	Complementary feeding method:						
	TSF	0.87 (0.60–1.26)	1.19 (0.83–1.70)	1.13 (0.79–1.62)	1.26 (0.75–2.10)	0.97 (0.70–1.34)	0.92 (0.66–1.27)
	Mixed	1	1	1	1	1	1
	BLW	0.61 (0.35–1.05)	0.70 (0.39–1.25)	0.69 (0.38–1.24)	0.74 (0.39–1.40)	0.89 (0.56–1.43)	0.81 (0.51–1.31)
	Types of CF:						
	Commercial	1.15 (0.65–2.04)	0.82 (0.48–1.38)	0.94 (0.55–1.59)	1.39 (0.66–2.95)	0.78 (0.48–1.26)	0.84 (0.52–1.35)
	Homemade	0.82 (0.51–1.33)	0.73 (0.46–1.14)	0.81 (0.51–1.27)	0.79 (0.44–1.40)	0.81 (0.54–1.21)	0.81 (0.54–1.21)
	Family	1	1	1	1	1	1
	Currently BF:						
	No	1.51 (1.01–2.25) *	1.56 (1.03–2.37) *	1.63 (1.07–2.49) *	1.52 (0.78–2.94)	1.24 (0.74–2.09)	1.36 (0.80–2.29)
	Yes	1	1	1	1	1	1
	EBF duration:						
	Never BF	0.92 (0.46–1.82)	1.52 (0.75–3.09)	1.52 (0.74–3.09)	1.92 (0.62–5.93)	2.39 (1.16–4.95) *	2.30 (1.11–4.79) *
	Never EBF	1.47 (0.93–2.34)	1.46 (0.94–2.26)	1.31 (0.84–2.04)	0.76 (0.41–1.39)	1.53 (1.01–2.32) *	1.42 (0.94–2.16)
	1–3 months	0.44 (0.21–0.88) *	0.87 (0.42–1.78)	0.63 (0.30–1.34)	0.62 (0.25–1.55)	1.66 (0.90–3.06)	1.42 (0.77–2.63)
	4–5 months	0.92 (0.57–1.47)	1.00 (0.62–1.61)	0.93 (0.58–1.50)	0.79 (0.41–1.50)	1.53 (1.00–2.35) *	1.45 (0.94–2.22)
	6–7 months	1	1	1	1	1	1
	>7 months	1.81 (0.60–5.47)	1.67 (0.62–4.51)	1.78 (0.66–4.83)	0.62 (0.20–1.86)	1.47 (0.57–3.75)	1.51 (0.59–3.87)

aOR—adjusted odds ratio; AS—added salt; ASu—added sugar; AS&Su—added salt and sugar; BF—breastfeeding; BLW—baby-led weaning; CF—complementary foods; CFI—complementary feeding introduction; CI—confidence interval; CF—complementary foods; EBF—exclusive breastfeeding; GDP—Gross Domestic Product; TSF—traditional spoon-feeding; * *p* ≤ 0.05; ** *p* ≤ 0.01; *** *p* ≤ 0.001.

**Table 4:** Current toddlers’ dietary habits and maternal concerns about toddlers’ feeding according to the age group and use of added salt, sugar, and both salt and sugar in the Polish sample (*n* = 4110).

Variables	12–24 Months (*n* = 2680)						25–36 Months (*n* = 1430)					
	AS		ASu		AS&Su		AS		ASu		AS&Su	
	Yes 52.8%	No 47.2%	Yes 34.7%	No 65.3%	Yes 26.8%	No 73.2%	Yes 74.4%	No 25.6%	Yes 59.0%	No 41.0%	Yes 50.8%	No 49.2%
Dietary pattern:												
→Pro-health	35.1	54.6 ***	37	48.1 ***	31.6	48.9 ***	28.9	44.8 ***	32.6	40.9 ***	26.6	39.5 ***
→Western-like	35.3	14.2	38.6	18.3	43.3	18.8	55.4	35.5	42.5	22.4	59.8	40.5
→Non-eaters	29.6	31.1	24.3	33.5	25.1	32.3	15.8	19.7	24.9	36.8	13.6	20
Eating at least one meal with the family	97.5	97.1	98.4	96.7 **	98.2	97.0	98.3	97.3	99.2	99.3 **	98.6	97.4
Eating meals separately	50.2	49.4	46.7	51.5 *	47.2	50.8	51.6	44.8 *	35.2	41.5 *	52.6	47.0 *
Eating while watching TV ^1^	34.1	19.8 ***	32.9	24.4 ***	37.2	23.8 ***	49.3	36.3 ***	13.4	8.5 ***	51.5	40.3 ***
Eating during playing	9.9	9.1	11.7	8.3 **	10.9	9.0	12.3	12.6	12.7	11.9	12.3	12.5
None concern	70.8	76.8 ***	70.7	75.2 **	69.1	75.3 ***	68.4	76.2 **	67.6	74.4 **	65.8	75.1 ***
Toddler eats too little	17.6	13.9 **	17.3	15.1	18.9	14.7 **	14.8	11.5	14.6	12.9	15.8	11.9
Toddler eats too much	7.2	6.9	7.3	6.9	7.1	7.0	4.5	4.4	4.2	4.9	4.0	5.0
Toddler eats same thing for longer period	20.2	15.7 **	20.8	16.7 **	22.3	16.6 ***	25.8	21.9	26.1	23.0	27.4	22.2 *
Toddler does not eat vegetables	19.4	11.9 ***	18.6	14.4 **	20.6	14.1 ***	25.5	18.3 ***	26.0	20.3 **	27.1	20.0 **
Toddler does not eat novel foods	16.3	11.5 ***	16.0	13.0 *	17.4	12.8 **	27.6	21.6 *	28.6	22.5 **	29.9	22.2 ***

^1^ including smartphones and tablets; AS—added salt; ASu—added sugar; AS&Su—added salt and sugar; * *p* ≤ 0.05; ** *p* ≤ 0.01; *** *p* ≤ 0.001.

**Table 5:** Current toddlers’ dietary habits and maternal concerns about toddlers’ feeding according to the age group and use of added salt, sugar, and both salt and sugar in the Austrian sample (*n* = 1783).

Variables	12–24 Months (*n* = 941)						25–36 Months (*n* = 842)					
	AS		ASu		AS&Su		AS		ASu		AS&Su	
	Yes 74.8%	No 25.2%	Yes 27.7%	No 72.3%	Yes 26.8%	No 73.2%	Yes 87.4%	No 12.6%	Yes 45.8%	No 54.2%	Yes 44.7%	No 55.3%
Dietary pattern:												
→Pro-health	37.8	40.9 ***	32.6	40.9 ***	33.1	40.6 ***	28.8	38.7	24.6	34.6 ***	25	34.1 ***
→Western-like	32.4	14.8	42.5	22.4	42.2	22.8	54.6	42.5	60.9	46.5	60.9	46.8
→Non-eaters	29.8	44.3	24.9	36.8	24.7	36.7	16.6	18.9	14.5	18.9	14,1	19.1
Eating at least one meal with the family	99.4	98.7	99.2	99.3	99.2	99.3	99.5	0.0	99.7	99.3	99.7	99.4
Eating meals separately	38.2	44.3	35.2	41.5	35.5	41.3	34.2	31.1	34.5	33.3	34.6	33.3
Eating while watching TV ^1^	9.9	9.7	13.4	8.5 *	12.7	8.8	17.8	16.0	18.9	16.4	18.6	16.7
Eating during playing	7.5	8.4	5.7	8.5	5.6	8.6	7.1	11.3	8.0	7.2	8.0	7.3
None concern	62.8	62.9	59.4	64.1	69.6	63.6	62.9	56.6	61.7	62.5	62.2	62.0
Toddler eats too little	17.8	16.9	20.3	16.5	19.9	16.7	19.3	17.0	20.2	18.0	18.0	20.2
Toddler eats too much	3.3	0.8*	1.9	2.9	2.0	2.9	3.3	2.8	3.9	2.6	2.6	4.0
Toddler eats same thing for longer period	14.8	15.6	16.1	14.6	15.9	14.6	16.7	13.2	16.8	15.8	16.3	16.2
Toddler does not eat vegetables	11.6	11.4	12.6	11.2	12.4	11.3	14.7	10.4	16.1	12.5	12.7	16.0
Toddler does not eat novel foods	8.4	7.6	10.3	7.4	10.4	7.4	15.5	9.4	16.6	13.2	13.1	16.8

^1^ including smartphones and tablets; AS—added salt; ASu—added sugar; AS&Su—added salt and sugar; * *p* ≤ 0.05; ** *p* ≤ 0.01; *** *p* ≤ 0.001.

## Supplementary Materials

The following are available online at https://www.mdpi.com/1660-4601/17/14/5025/s1, Table S1: Univariate regression analysis models of factors influencing the use of added salt, sugar, and salt and sugar in Polish toddlers (*n* = 4110); Table S2: Univariate regression analysis models of factors influencing the use of added salt, sugar, and salt and sugar in Austrian toddlers (*n* = 1783); Table S3: Sources of knowledge about children nutrition according to the country, age group and use of added salt, sugar, and both salt and sugar.

## Abbreviations

aOR	Adjusted odds ratio
AS	Added salt
AS&Su	Added salt and sugar
ASu	Added sugar
BF	Breastfeeding
BLW	Baby led weaning
CF	Complementary foods
CFI	Complementary feeding introduction
CI	Confidence interval
DOHaD	Developmental Origins of Health and Disease hypothesis
EBF	Exclusive breastfeeding
EFSA	European Food Safety Authority
GDP	Gross domestic product
OR	Odds ratio
TSF	Traditional spoon-feeding
