# Supplementary material for: The Use of Added Salt and Sugar in the Diet of Polish and Austrian Toddlers. Associated Factors and Dietary Patterns, Feeding and Maternal Practices
Source: Int J Environ Res Public Health. 2020 Jul 13;17(14):5025. doi: 10.3390/ijerph17145025 (PMC7400520; doi:10.3390/ijerph17145025)
Supplement: Supplementary file 1 [file ijerph-17-05025-s001.pdf]

International Journal of *Environmental Research and Public Health*

MDPI

Article

The Use of Added Salt and Sugar in the Diet of Polish and Austrian Toddlers. Associated Factors and Dietary Patterns, Feeding and Maternal Practices

Daria Masztalerz-Kozubek [1], Monika A. Zielinska [1], Petra Rust [2], Dorota Majchrzak [2] and Jadwiga Hamulka [1,*]

Table S1. Univariate regression analysis models of factors influencing the use of added salt, sugar, and salt and sugar in Polish toddlers (n = 4110).

	Variable	12 - 24 months (n = 2680)			25 - 36 months (n = 1430)		
		**AS** OR (95% CI)	**ASu** OR (95% CI)	**AS&Su** OR (95% CI)	**AS** OR (95% CI)	**ASu** OR (95% CI)	**AS&Su** OR (95% CI)
SOCIODEMOGRAPHIC FACTORS	**Toddler age:**	1.18 (1.16 – 1.21)***	1.14 (1.11 – 1.16)***	1.16 (1.14 – 1.19)***	1.02 (0.99 – 1.06)	1.02 (0.99 – 1.05)	1.01 (0.98 – 1.04)
	**Maternal age:**						
	< 25 years	0.88 (0.62 – 1.24)	1.27 (0.89 – 1.81)	1.13 (0.77 – 1.65)	1.54 (0.76 – 3.14)	2.38 (1.22 – 4.63)**	2.28 (1.24 – 4.22)**
	25–29 years	0.93 (0.79 – 1.10)	1.03 (0.87 – 1.23)	1.04 (0.86 – 1.26)	1.38 (1.03 – 1.85)*	1.45 (1.12 – 1.89)**	1.46 (1.13 – 1.88)**
	30–34 years	1	1	1	1	1	1
	35–39 years	1.06 (0.83 – 1.35)	0.81 (0.62 – 1.06)	0.85 (0.64 – 1.13)	1.29 (0.92 – 1.82)	0.95 (0.71 – 1.28)	1.10 (0.82 – 1.47)
	≥ 40 years	0.73 (0.37 – 1.43)	0.24 (0.08 – 0.69)**	0.35 (0.12 – 1.00)*	1.45 (0.58 – 3.62)	0.67 (0.31 – 1.42)	0.83 (0.39 – 1.78)
	**Maternal education:**						
	Primary and vocational	2.41 (1.06 – 5.46)*	1.81 (0.87 – 3.77)	2.67 (1.28 – 5.56)**	3.28 (0.41 – 25.98)	1.65 (0.43 – 6.42)	2.33 (0.60 – 9.06)
	High school	1.16 (0.91 – 1.46)	1.19 (0.94 – 1.52)	1.30 (1.01 – 1.68)*	1.69 (1.10 – 2.59)*	1.13 (0.80 – 1.58)	1.26 (0.90 – 1.75)
	University	1	1	1	1	1	1
	**Parity:**						
	Primiparias	1	1	1	1	1	1
	Multiparias	1.81 (1.52 – 2.16)***	1.34 (1.13 – 1.60)***	1.54 (1.28 – 1.85)***	1.41 (1.11 – 1.80)**	1.30 (1.05 – 1.61)**	1.36 (1.10 – 1.68)**
	**Living area:**						
	Rural	1.14 (0.93 – 1.39)	1.22 (0.99 – 1.49)	1.23 (0.99 – 1.53)	1.20 (0.86 – 1.66)	1.34 (1.01 – 1.79)*	1.34 (1.02 – 1.77)*
	Urban	1	1	1	1	1	1
	**Macroeconomic region (GDP EU-28 average):**						
	47–50%	1.12 (0.87 – 1.45)	0.95 (0.72 – 1.24)	0.93 (0.70 – 1.24)	1.00 (0.66 – 1.51)	1.18 (0.82 – 1.70)	1.11 (0.77 – 1.59)
	51–100%	0.98 (0.81 – 1.18)	1.03 (0.85 – 1.25)	0.94 (0.77 – 1.16)	0.98 (0.73 – 1.30)	1.14 (0.89 – 1.47)	1.13 (0.88 – 1.45)
	101–110%	1	1	1	1	1	1
	**Average monthly income per capita (PLN):**						

	<500	1.68 (0.85 – 3.33)	1.09 (0.54 – 2.22)	1.86 (0.91 – 3.83)	2.51 (0.71 – 8.85)	2.27 (0.80 – 6.40)	2.56 (0.96 – 6.82)
	500–1000	1.37 (1.02 – 1.85)*	1.41 (1.03 – 1.92)*	1.75 (1.25 – 2.47)***	1.31 (0.83 – 2.07)	1.05 (0.70 – 1.58)	1.12 (0.75 – 1.67)
	1001–2000	1.17 (0.90 – 1.51)	1.17 (0.88 – 1.54)	1.33 (0.98 – 1.82)	1.13 (0.77 – 1.65)	0.93 (0.66 – 1.31)	1.00 (0.72 – 1.41)
	2001–2500	1.43 (1.07 – 1.91)**	1.34 (0.99 – 1.81)	1.68 (1.20 – 2.34)**	1.23 (0.80 – 1.89)	1.03 (0.70 – 1.52)	1.02 (0.69 – 1.49)
	2501–3000	1	1	1	1	1	1
	≥3001	0.98 (0.74 – 1.28)	0.95 (0.71 – 1.28	1.11 (0.79 – 1.55)	1.11 (0.74 – 1.66)	0.83 (0.58 – 1.20)	0.82 (0.57 – 1.17)
**FEEDING-RELATED FACTORS**	**Timing of CFI:**						
	<4 months	1.53 (0.87 – 2.67)	1.30 (0.74 – 2.29)	1.24 (0.67 – 2.29)	1.98 (0.94 – 4.19)	0.83 (0.46 – 1.50)	1.06 (0.59 – 1.91)
	4-6 months	1.36 (1.16 – 1.59)***	1.08 (0.91 – 1.27)	1.25 (1.04 – 1.49)*	1.48 (1.16 – 1.90)**	1.26 (1.01 – 1.58)*	1.40 (1.12 – 1.75)**
	≥7 months	1	1	1	1	1	1
	**Complementary feeding method:**						
	Spoon feeding	1.45 (1.21 – 1.74)***	0.99 (0.82 – 1.19)	1.14 (0.93 – 1.38)	1.15 (0.87 – 1.53)	1.09 (0.85 – 1.39)	1.12 (0.88 – 1.42)
	Mixed	1	1	1	1	1	1
	BLW	0.70 (0.57 – 0.87)***	1.00 (0.80 – 1.24)	0.82 (0.64 – 1.05	0.70 (0.52 – 0.95)*	0.67 (0.50 – 0.88)**	0.66 (0.50 – 0.88)**
	**Types of CF:**						
	Commercial	1.59 (1.28 – 1.97)***	0.87 (0.70 – 1.09)	1.09 (0.85 – 1.39)	0.96 (0.69 – 1.34)	0.97 (0.73 – 1.30)	1.02 (0.77 – 1.36)
	Homemade	1.22 (1.02 – 1.45)*	0.96 (0.80 – 1.16)	1.14 (0.94 – 1.40)	0.99 (0.75 – 1.30)	0.99 (0.78 – 1.27)	0.98 (0.77 – 1.24)
	Family	1	1	1	1	1	1
	**Currently BF:**						
	No	1.77 (1.51 – 2.07)***	1.30 (1.11 – 1.53)**	1.48 (1.25 – 1.77)***	1.51 (1.17 – 1.95)**	1.07 (0.85 – 1.35)	1.20 (0.95 – 1.51)
	Yes	1	1	1	1	1	1
	**EBF duration:**						
	Never BF	1.35 (0.94 – 1.93)	1.61 (1.13 – 2.31)**	1.60 (1.09 – 2.33)*	2.51 (1.31 – 4.81)**	1.34 (0.82 – 2.21)	1.51 (0.93 – 2.46)
	Never EBF	1.06 (0.89 – 1.25)	1.03 (0.86 – 1.23)	1.07 (0.88 – 1.30)	1.34 (1.02 – 1.75)*	1.15 (0.90 – 1.47)	1.21 (0.95 – 1.54)
	1-3 months	2.00 (1.10 – 3.62)*	0.94 (0.51 – 1.72)	1.41 (0.77 – 2.60)	1.52 (0.67 – 3.44)	1.04 (0.52 – 2.08)	1.36 (0.68 – 2.71)
	4-5 months	2.08 (1.52 – 2.86)***	1.80 (1.33 – 2.44)***	1.98 (1.45 – 2.71)***	1.97 (1.26 – 3.08)**	1.21 (0.83 – 1.76)	1.38 (0.95 – 1.98)
	6-7 months	1	1	1	1	1	1
	>7 months	0.80 (0.21 – 2.99)	0.26 (0.03 – 2.06)	0.39 (0.05 – 3.11)	0.79 (0.23 – 2.74)	0.93 (0.28 – 3.11)	0.95 (0.29 – 3.17)

AS – added salt; ASu – added sugar; AS&Su – added salt and sugar; BF – breastfeeding; BLW – Baby-Led Weaning; CF – complementary foods; CFI – complementary feeding introduction; CI – confidence interval; CF – complementary foods; EBF – exclusive breastfeeding; GDP – Gross Domestic Product; OR – odds ratio; TSF – traditional spoonfeeding; * $p \leq 0.05$; ** $p \leq 0.01$; *** $p \leq 0.001$.

Table S2. Univariate regression analysis models of factors influencing the use of added salt, sugar, and salt and sugar in Austrian toddlers (*n* = 1783).

	Variable	12 - 24 months (*n* = 941)			25 - 36 months (*n* = 842)		
		**AS** OR (95% CI)	**ASu** OR (95% CI)	**AS&Su** OR (95% CI)	**AS** OR (95% CI)	**ASu** OR (95% CI)	**AS&Su** OR (95% CI)
**SOCIODEMOGRAPHIC FACTORS**	**Toddler age:**	1.16 (1.11 – 1.21)***	1.13 (1.09 – 1.18)***	1.14 (1.09 – 1.18)***	1.02 (0.97 – 1.08)	1.04 (1.00 – 1.08)*	1.05 (1.01 – 1.09)*
	**Maternal age:**						
	< 25 years	1.56 (0.78 – 3.11)	1.17 (0.65 – 2.12)	1.24 (0.69 – 2.23)	0.58 (0.19 – 1.81)	0.72 (0.30 – 1.70)	0.61 (0.25 – 1.49)
	25–29 years	1.08 (0.75 – 1.56)	0.99 (0.70 – 1.40)	0.97 (0.68 – 1.38)	0.77 (0.45 – 1.31)	0.87 (0.61 – 1.24)	0.88 (0.62 – 1.26)
	30–34 years	1	1	1	1	1	1
	35–39 years	0.97 (0.66 – 1.42)	0.85 (0.58 – 1.25)	0.85 (0.58 – 1.26)	0.85 (0.51 – 1.42)	0.91 (0.65 – 1.27)	0.88 (0.63 – 1.23)
	≥ 40 years	0.89 (0.40 – 2.00)	0.99 (0.44 – 2.21)	1.05 (0.47 – 2.34)	0.64 (0.29 – 1.42)	1.11 (0.63 – 1.96)	1.07 (0.61 – 1.89)
	**Maternal education:**						
	Primary and vocational	1.28 (0.90 – 1.80)	1.32 (0.96 – 1.81)	1.34 (0.98 – 1.85)	1.01 (0.63 – 1.61)	1.06 (0.77 – 1.45)	1.01 (0.74 – 1.39)
	High school	0.51 (0.25 – 1.06)	0.78 (0.33 – 1.84)	0.68 (0.28 – 1.69)	2.21 (0.52 – 9.44)	1.57 (0.77 – 3.21)	1.43 (0.70 – 2.91)
	University	1	1	1	1	1	1
	**Parity:**						
	Primiparias	1	1	1	1	1	1
	Multiparias	1.48 (1.09 – 2.01)**	1.82 (1.37 – 2.43)***	1.82 (1.36 – 2.44)***	0.85 (0.55 – 1.30)	1.10 (0.83 – 1.45)	1.13 (0.85 – 1.49)
	**Living area:**						
	Rural	1.47 (1.10 – 1.98)**	1.72 (1.28 – 2.31)***	1.73 (1.28 – 2.34)***	1.16 (0.77 – 1.75)	1.23 (0.94 – 1.62)	1.20 (0.91 – 1.58)
	Urban	1	1	1	1	1	1
	**Macroeconomic region (GDP EU-28 average):**						
	47–50%	-	-	-	-	-	-
	51–100%	1.18 (0.58 – 2.38)	1.43 (0.78 – 2.60)	1.48 (0.81 – 2.70)	3.55 (0.83 – 15.12)	0.38 (0.19 – 0.74)**	0.39 (0.20 – 0.76)**
	101–110%	1	1	1	1	1	1
	111–130%	0.83 (0.57 – 1.19)	1.20 (0.85 – 1.69)	1.15 (0.81 – 1.63)	1.11 (0.68 – 1.83)	0.81 (0.58 – 1.14)	0.76 (0.54 – 1.06)
	131–150%	1.01 (0.58 – 1.75)	0.64 (0.36 – 1.13)	0.61 (0.34 – 1.10)	2.07 (0.91 – 4.75)	0.64 (0.41 – 1.02)	0.63 (0.40 – 1.00)
	>150%	0.66 (0.44 – 1.01)	0.85 (0.55 – 1.32)	0.82 (0.53 – 1.28)	0.97 (0.55 – 1.71)	0.58 (0.39 – 0.86)**	0.56 (0.37 – 0.83)*
	**Average monthly income per capita (EUR):**						

	<1000	0.91 (0.45 – 1.81)	0.97 (0.47 – 1.99)	1.19 (0.57 – 2.51)	1.42 (0.62 – 3.23)	0.97 (0.53 – 1.77)	0.90 (0.49 – 1.65)
	1000-1500	1.22 (0.68 – 2.21)	1.51 (0.84 – 2.72)	1.66 (0.90 – 3.07)	2.26 (1.12 – 4.58)*	1.19 (0.74 – 1.93)	1.18 (0.73 – 1.91)
	1501-2000	1.24 (0.71 – 2.15)	1.00 (0.57 – 1.77)	1.24 (0.69 – 2.25)	1.51 (0.82 – 2.78)	1.15 (0.73 – 1.81)	1.15 (0.73 – 1.82)
	2001–3000	1.07 (0.61 – 1.87)	1.18 (0.66 – 2.09)	1.40 (0.77 – 2.55)	1.67 (0.88 – 3.17)	1.10 (0.69 – 1.75)	1.10 (0.69 – 1.76)
	3001-5000	1	1	1	1	1	1
	≥5001	1.04 (0.30 – 3.62)	1.50 (0.46 – 4.92)	1.85 (0.56 – 6.15)	1.65 (0.45 – 6.05)	1.43 (0.60 – 3.42)	1.26 (0.53 – 3.02)
**FEEDING-RELATED FACTORS**	**Timing of CFI:**						
	<4 months	1.11 (0.51 – 2.43)	0.85 (0.39 – 1.87)	0.76 (0.34 – 1.71)	1.85 (0.61 – 5.60)	1.21 (0.60 – 2.45)	1.00 (0.49 – 2.04)
	4-6 months	1.13 (0.79 – 1.62)	0.99 (0.69 – 1.40)	0.96 (0.67 – 1.36)	1.70 (1.07 – 2.71)*	1.02 (0.73 – 1.44)	1.00 (0.71 – 1.41)
	≥7 months	1	1	1	1	1	1
	**Complementary feeding method:**						
	Spoon feeding	0.89 (0.64 – 1.22)	1.10 (0.81 – 1.49)	1.06 (0.78 – 1.45)	1.25 (0.78 – 2.02)	1.04 (0.77 – 1.40)	0.99 (0.73 – 1.33)
	Mixed	1	1	1	1	1	1
	BLW	0.60 (0.39 – 0.92)*	0.71 (0.44 – 1.13)	0.66 (0.41 – 1.08)	0.63 (0.37 – 1.08)	0.83 (0.55 – 1.24)	0.77 (0.51 – 1.15)
	**Types of CF:**						
	Commercial	1.33 (0.86 – 2.06)	1.08 (0.72 – 1.62)	1.17 (0.78 – 1.77)	2.05 (1.08 – 3.87)*	0.99 (0.67 – 1.46)	1.03 (0.70 – 1.51)
	Homemade	1.00 (0.70 – 1.41)	0.90 (0.64 – 1.28)	0.96 (0.68 – 1.36)	1.12 (0.70 – 1.79)	0.93 (0.67 – 1.30)	0.93 (0.67 – 1.30)
	Family	1	1	1	1	1	1
	**Currently BF:**						
	No	1.88 (1.39 – 2.53)***	1.73 (1.27 – 2.35)***	1.72 (1.26 – 2.35)***	1.13 (0.69 – 1.88)	1.01 (0.72 – 1.43)	1.08 (0.76 – 1.53)
	Yes	1	1	1	1	1	1
	**EBF duration:**						
	Never BF	0.89 (0.50 – 1.58)	1.20 (0.67 – 2.12)	1.20 (0.67 – 2.12)	1.92 (0.77 – 4.79)	1.89 (1.13 – 3.16)*	1.71 (1.02 – 2.86)*
	Never EBF	1.41 (0.94 – 2.10)	1.29 (0.88 – 1.88)	1.19 (0.81 – 1.74)	1.08 (0.63 – 1.85)	1.52 (1.05 – 2.20)*	1.42 (0.98 – 2.06)
	1-3 months	0.71 (0.39 – 1.31)	1.19 (0.63 – 2.24)	0.91 (0.47 – 1.78)	1.07 (0.46 – 2.46)	1.57 (0.90 – 2.76)	1.38 (0.79 – 2.43)
	4-5 months	1.00 (0.67 – 1.50)	1.00 (0.66 – 1.51)	0.96 (0.63 – 1.45)	1.14 (0.65 – 1.99)	1.45 (0.99 – 2.12)	1.40 (0.96 – 2.05)
	6-7 months	1	1	1	1	1	1
	>7 months	1.38 (0.50 – 3.85)	1.47 (0.60 – 3.60)	1.47 (0.60 – 3.60)	0.41 (0.15 – 1.14)	1.35 (0.56 – 3.27)	1.35 (0.56 – 3.27)

AS – added salt; ASu – added sugar; AS&Su – added salt and sugar; BF – breastfeeding; BLW – Baby-Led Weaning; CF – complementary foods; CFI – complementary feeding introduction; CI – confidence interval; CF – complementary foods; EBF – exclusive breastfeeding; GDP – Gross Domestic Product; OR – odds ratio; TSF – traditional spoonfeeding;

* $p \leq 0.05$; ** $p \leq 0.01$; *** $p \leq 0.001$.

Table S3. Sources of knowledge about children nutrition according to the country, age group and use of added salt, sugar, and both salt and sugar.

	**Variable**	**12 - 24 months**						**25 - 36 months**					
		**AS**		**ASu**		**AS&Su**		**AS**		**ASu**		**AS&Su**	
		**Yes**	**No**	**Yes**	**No**	**Yes**	**No**	**Yes**	**No**	**Yes**	**No**	**Yes**	**No**
**Poland**	**Internet**	93.1	94.3	93.9	93.6	93.5	93.8	90.9	88.8	88.4	91.7*	92.1	88.5*
	**Books**	62.2	68.5***	64.2	65.7	62.0	66.3*	62.6	72.4***	69.2	62.3**	61.2	69.2**
	**Magazines**	14.3	11.4*	13.0	12.8	13.9	12.5	15.8	16.7	14.3	17.2	17.8	14.2
	**TV**	4.7	2.0***	4.5	2.8*	5.2	2.8**	3.6	3.6	3.9	3.3	3.4	3.7
	**Family or friends**	49.9	40.6***	49.6	43.3**	53.2	42.7***	47.1	39.9**	40.5	48.5**	49.9	40.5***
	**Doctor or midwife**	32.0	27.7	31.6	29.1	32.5	29.1	34.7	32.5	33.4	34.6	35.0	33.2
	**Nutritionist or dietitian**	11.4	15.8***	12.2	14.2	10.7	14.5**	8.2	11.5	10.4	8.1	7.9	10.2
**Austria**	**Internet**	85.2	86.5	85.1	85.7	84.9	85.8	80.7	81.1	81.4	80.1	81.3	80.1
	**Books**	80.7	79.7	76.6	81.9	76.5	81.9	80.7	83.0	82.0	79.8	81.8	80.1
	**Magazines**	26.8	27.0	26.8	26.9	25.9	27.2	29.9	36.8	30.9	30.6	31.3	30.1
	**TV**	8.1	7.6	8.4	7.8	8.0	8.0	7.5	11.3	7.0	9.1	7.5	8.5
	**Family or friends**	82.4	77.6	86.2	79.3*	87.3	79.0**	77.3	71.7	74.3	79.3	74.7	79.0
	**Doctor or midwife**	65.5	63.7	71.6	62.5**	71.7	62.6**	67.1	59.4	63.2	69.7*	63.1	69.9*
	**Nutritionist or dietitian**	36.8	50.2***	33.3	42.8**	33.1	42.8**	35.6	48.1**	39.3	34.7	39.7	34.0

AS – added salt; ASu – added sugar; AS&Su – added salt and sugar; * $p \le 0.05$; ** $p \le 0.01$; *** $p \le 0.001$.
